# Adequate Care Coordination is Associated with Reduced Unmet Mental Health Needs for Children

**DOI:** 10.21203/rs.3.rs-7906988/v1

**Published:** 2025-11-20

**Authors:** Genevieve Graaf, Kristin Gigli, Phillip Hughes, Kathleen Thomas

**Affiliations:** The University of Texas at Arlington; The University of Texas at Arlington; University of North Carolina at Chapel Hill; University of North Carolina at Chapel Hill

**Keywords:** child mental health, youth mental health, care coordination, school mental health

## Abstract

Children with special healthcare needs (CSHCN), especially those with mental health needs, often receive services from multiple service sectors, including schools. Thus, care coordination may play a critical role in access to mental health care for these children. This pooled cross-sectional study used multivariable logistic regression with data from the National Survey of Children’s Health (2020–2023), focusing on a subsample of children with caregiver-reported mental health problems and need for mental healthcare. It estimates the prevalence of parent-reported need for and receipt of effective care coordination and provider communication with schools. It also measures the association between parent-reported receipt of these services with unmet need for mental healthcare. Most caregivers reported need for care coordination (51.3%) and provider communication with schools (60.7%). Among those with need, 48.3% reported not having their care coordination needs met, and 57.2% of families reported unmet need for provider communication with schools. The probability of reported unmet mental health needs was 12 percentage points lower when children had all needs for care coordination met and 10 points lower when providers communicated with schools. Results suggest that having care coordination needs met and provider communication with schools may play an important role in children accessing needed mental healthcare.

## Introduction

Children with special healthcare needs (CSHCN), especially those with mental health needs, often receive care from multiple service sectors (e.g., public health, schools, health systems) to address their complex health concerns (Hansen et al., 2004). Families who receive professional assistance coordinating care across providers are more likely to access needed specialist care,(K. Miller, 2014) assistive technologies, and specialized therapies (Graaf & Gigli, 2022) than those who do not. For this reason, assistance in care coordination across services is highly valued by caregivers (Graaf et al., 2024) and can play a critical role in access to adequate care for these children (Lindly et al., 2020). In particular, caregivers of children with mental health needs cite the importance of provider communication with schools—where children spend the majority of their time and where classroom accommodations and school-based supports are particularly important for their success (Graaf et al., 2024). However, compared to children with other types of complex healthcare needs, children with mental health problems are less likely to receive effective care coordination (Miller et al., 2018) and providers are less likely to communicate with their schools (Geffel et al., 2022).

Nationwide, little is known about the prevalence of care coordination and provider-school communication for children with mental health problems. A lone study, drawing from 2007 data, demonstrated that over 40% of children with mental health conditions had unmet need for care coordination. Further, though evidence suggests that receipt of these services is associated with increased access to specialized medical care (Graaf & Gigli, 2022; Miller, 2014), yet, the role that care coordination activities may play in mental healthcare access is unknown. For children with caregiver-reported mental health problems and need for mental healthcare, this study draws on updated data (2020–2023) to 1) assess the prevalence of parent-reported need for and receipt of adequate care coordination and provider communication with schools and 2) estimate the association between parentreported receipt of these services and unmet need for mental healthcare.

## Methods

**Figure 1:**
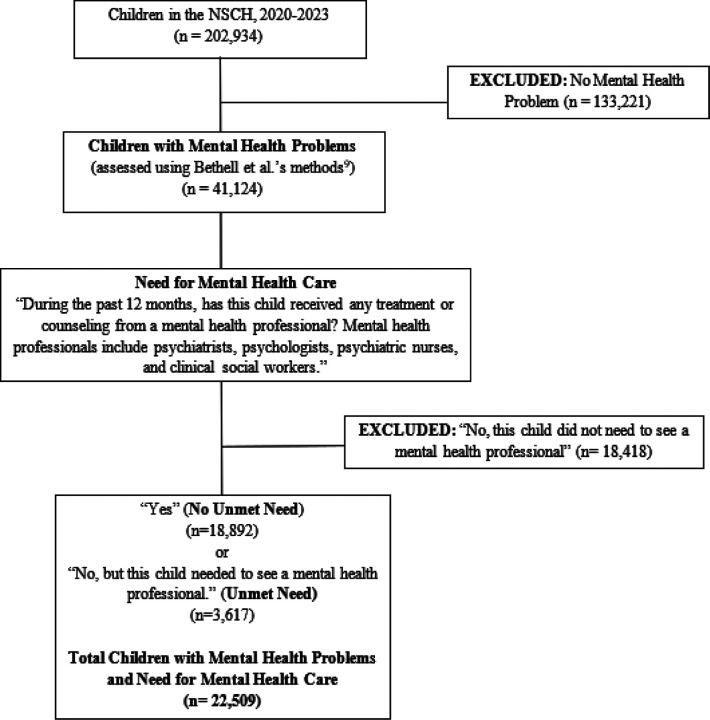
Children with Mental Health Problems and Need for Mental Health Care: Study Sampling and Sample Sizes

We conducted a pooled cross-sectional study, pooling data from the National Survey of Children’s Health (NSCH; 2020–2023). Information about the NSCH methodology is detailed elsewhere (Ghandour et al., 2018). The sample included children (age 0–17) with parent-reported mental health problems, identified through a either a caregiver-reported child emotional, behavioral, or developmental problem on the Children with Special Health Care Needs Screener subscale within the NSCH, or a caregiver’s report of their child’s receipt of a mental health diagnosis (anxiety, depression, attention-deficit hyperactivity disorder, or behavioral or conduct problems) (Bethell et al., 2015). Caregivers were asked the following question: “During the past 12 months, has this child received any treatment or counseling from a mental health professional? Mental health professionals include psychiatrists, psychologists, psychiatric nurses, and clinical social workers.” Children whose caregiver reported “Yes” or “No” to this question were coded as having need for mental health care and were included in the sample. Children whose caregiver responded, “No, this child did not need to see a mental health professional,” were excluded from the sample as they indicated no need for mental health care. (See Fig. 1 in the online Supplement) The outcome variable, unmet need for mental health care, was also constructed from this variable. Consistent with several other studies, children with mental health problems whose caregivers reported that they needed to see a mental health professional in the past 12 months, but did not see one were coded as having unmet mental health need (Casseus, 2023; Lebrun-Harris et al., 2019; Pasli & Tumin, 2022).

**Figure 2:**
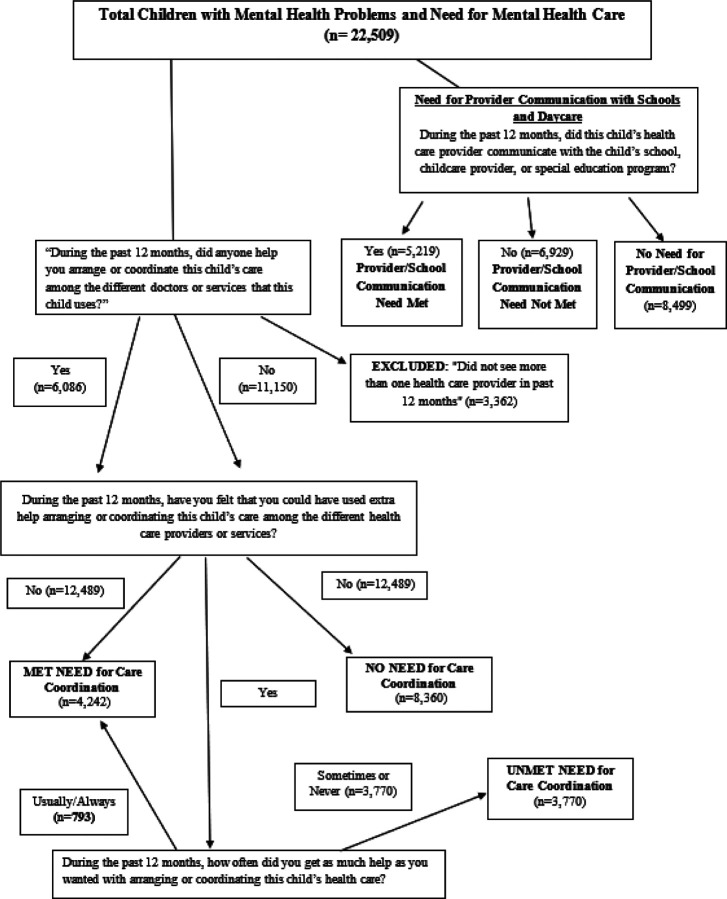
Care Coordination and Provider/School Communication Need and Unmet Need: Study Sampling and Sample Sizes

Analysis addressed two predictors of unmet mental health need: 1) having care coordination needs met, and 2) provider communication with a child’s school or childcare. Need, met need, and unmet need for care coordination were structured according to methods developed by Brown and colleagues (see Fig. 2 in the online supplement).(Brown et al., 2014) Caregivers of children were asked a series of questions about their need for coordination of healthcare services. The first was: “During the past 12 months, did anyone help you arrange or coordinate care among the different doctors or services that this child uses?” Children whose caregivers responded “Did not see more than one healthcare provider in the past 12 months” were excluded from the sample.

Caregivers remaining in the sample were asked the following two questions: “During the past 12 months, have you felt you could have used extra help arranging or coordinating this child’s care among the different healthcare providers or services?”; and “During the past 12 months, how often did you get as much help as you wanted with arranging or coordinating care?” We created two binary variables to measure care coordination need and unmet need (See Fig. 1). Caregivers reporting that no one helped them arrange or coordinate their child’s care and that they did not need extra help were categorized as having no need for care coordination. Having all care coordination needs met was defined as either 1) receiving help arranging or coordinating care and not needing any extra help, or 2) needing extra help and usually or always receiving as much extra help as they wanted. Unmet need for care coordination was defined as sometimes or never receiving as much help in arranging or coordinating care as was wanted.

Provider communication with schools is derived from a question asking caregivers, for all children regardless of how many healthcare providers or doctors they had seen in the past 12 months, if their child’s healthcare provider had communicated with their child’s school, childcare provider, or special education program. Children whose caregivers reported “yes” were coded as needing provider communication with schools and as having received that service. Children whose caregivers reported “no” were coded as needing provider communication with their child’s school but not receiving it. Children whose caregivers responded that their child “did not need” that service were coded as not needing provider communication with schools.

Univariate analysis described rates of parent-reported unmet need for mental healthcare and need and unmet need for care coordination and provider communication with schools or childcare. Separate logistic regression models estimated associations between each predictor variable and parent-reported unmet mental health need, each using separate subsamples. For effective care coordination, only children whose caregiver reported seeing more than 2 providers in the last year were included in the subsample. For provider communication with schools, only those whose caregiver reported need for providers to communication with schools were included in the subsample. Regression analyses controlled for child and family covariates, drawn from the same dataset, guided by the Behavioral Model of Health Service Use.(Andersen, 1995) Low missingness supported a complete-case approach for analysis,(Langkamp et al., 2010) resulting in slightly smaller sample sizes after inclusion of covariates.

Calculation of odds ratios incorporates unobserved study and modeling error into the denominator of the calculation, making odds ratios relatively ungeneralizable.(Norton et al., 2024) For this reason, average marginal effects, which are more transferrable across study designs, were generated in marginal post-estimation for each predictor variable.(Norton et al., 2024) Analyses were conducted using STATA v.16.1 (StataCorp, College Station, TX), employing the survey (*‘svy’* and *‘subpop’*) commands to account for survey weighting, stratification, and clustering to generate nationally representative results.

Data used in this study spanned the COVID-19 pandemic which created changes in children’s need for mental health care and practices in mental health service delivery (Kauhanen et al., 2023; Palinkas et al., 2021; Purtle et al., 2022; Sklar et al., 2021; Zablotsky et al., 2022). To assess if pandemic impacts were observable in our data, and would thus contribute to inaccurate assessments of usual care circumstances, analysis was conducted using the National Survey of Children’s Health pooling data from 2016 to 2019. Because results were nearly identical to those generated using 2020–2023 data, results from analysis with the most recent data are reported here.

## Results

Weighted proportions of need and unmet need for mental health care, care coordination, and provider communication with schools or childcare are reported in Table 1. For children with parent-reported mental health problems and need for mental healthcare, 18.6% reported unmet mental health need. Half (51.3%) reported need for care coordination and 60.7% reported need for providers to communicate with their child’s school or childcare. Almost half of caregivers needing care coordination (48.3%) reported not having all their care coordination needs met, with 57.2% of families needing provider communication with schools reporting that providers failed to do so. In logistic regression, the probability of unmet mental health needs was 12 percentage points lower when caregivers had all their care coordination needs met [Average Marginal Effect (AME) = −0.12 (−0.16, −0.08)] and 10 percentage points lower when providers communicated with school or childcare [AME = −0.11 (−0.13, −0.07)]. See online supplement for sample description and full models.

## Discussion

Among children with mental health problems, potential need for care coordination and provider/school communication is high. However, most families did not receive effective care coordination and experienced poor provider communications with schools. Results suggest that effective care coordination and provider communication with schools, may play an important role in ensuring these children access needed mental health services. These activities improve coordination and communication across service sectors which is deeply valued by caregivers of children with mental health concerns (Graaf et al., 2024), and this study suggests they are also associated with improved access to needed mental healthcare.

These findings are not without limitations. First, the NSCH excludes children in institutional settings, including justice or psychiatric institutions, among whom more complex mental health needs are overrepresented. Further, the study is limited by the data collected in the NSCH, as well as the way in which certain constructs were measured in the NSCH. Covariates were limited to individual characteristics and factors included in the dataset, and measures were based on caregiver self-report. Thus, indicators or mental health problems or use of mental health services were not assessed using validated clinical measures which, together with recall bias may result in inaccurate reports of unmet mental health need. Finally, the measure of unmet need was based on caregivers’ report regarding whether their child saw a mental health provider in the last twelve months. This measure may exclude children whose mental health needs were met by their primary care provider and children who saw a mental health provider but whose mental health needs were not entirely met.

However, given rising concerns about children’s increasing need for mental health care (Foy et al., 2019), study results may have important implications for pediatric primary care and specialist practices (Gigli & Graaf, 2025). Families often are unaware of where to access mental health care and schools can be important in connecting children with mental health services, either by providing them directly in the school settings or referring families to community-based supports (Hoover et al., 2020). The American Academy of Pediatrics reaffirmed the Mental Health Competencies for Pediatric Practice in 2025, which emphasizes the importance of pediatricians engaging a child’s schools to appropriately address their mental health needs (Foy et al., 2019). Provider communication with schools or childcare can alert school personnel to the need for care, provide a pathway for schools to alert the pediatrician to mental health needs, and can support the coordination of that care to ensure that schools are fully informed about medication use or behavioral or emotional support plans.

However, many challenges to adequately supporting care coordination and school communication exist in pediatric practices. These include inadequate reimbursement for care coordination activities, weak partnerships or connections to community-based or school providers, inadequate mental health workforce in the community or schools, and insufficient pediatric care staffing and expertise to address more complex mental health needs (Foy et al., 2019). However, structured and routine communication processes—such as regularly scheduled care team meetings or check-ins—and the support of healthcare social workers can assist in overcoming some of these challenges (Ross et al., 2019). To support these efforts in pediatric practices and other community-based behavioral health providers, attention to increasing time and compensation for pediatric care coordination activities is needed (Cheng & Perrin, 2024; The Future Pediatric Subspecialty Physician Workforce, 2023). State mental health agencies, Medicaid authorities, and insurance market governing bodies can play an important role in the extent to which these services are covered for children in their state and the adequacy of reimbursement rates for these services. Public concern regarding growing mental health need may be leveraged to encourage state administrations to expand coverage and increase payment for care coordination activities. Such policy actions may play a pivotal role in expanding access to needed mental health care for children.

## Supplementary Material

Supplementary Files

This is a list of supplementary files associated with this preprint. Click to download.

- CCUnmetMHNeedsSupplementFinal10.15.251.docx

## Figures and Tables

**Table 1:** Mental Health Care and Care Coordination Among Children with Mental Health Problems & Need for Mental Health Care (n = 22,509)

	n	%*
**Unmet Need for Mental Health Care**		
No unmet need mental health care	18,892	81.4%
Unmet need mental health care	3,617	18.6%
**Need for Care Coordination**		
Had need for care coordination	8,732	51.3%
No need for care coordination	8,360	48.7%
**Unmet Need Care Coordination** **		
Unmet need for care coordination	3,770	48.3%
No unmet need for care coordination	4,242	51.7%
**Need for Doctor Communication with Schools and Childcare**		
No school communication needed	8,499	39.3%
Doctor/school communication needed	12,148	60.7%
**Doctor Communicates with Schools and Childcare** **		
Doctors did not communicate with school or childcare	6,929	57.2%
Doctors communicate with child’s school or childcare	5,219	42.8%

Data Source: National Survey of Children’s Health (NSCH), 2020–2023;

* Weighted Proportions;

** Among those with need for care coordination or provider communication with schools or childcare

Alternative text: 18.6% of children and youth with parent-reported mental health problems and need for mental healthcare reported unmet mental health need. Over half of caregivers reported need for care coordination, with just under half of families having all their care coordination needs met. Over 60% of families reported need for providers to communicate with their child’s school or childcare but almost 60% of these families reported that providers failed to do so.

**Table 2:** Association between Care Coordination and Mental Health Service Use among Children with Mental Health Problems and Need for Mental Health Care

	AME	p<	95%	
Care Coordination Need Met** n = 21,762*	−0.12	<0.001	−0.16	−0.08
**Provider Communicated with Schools or Daycare** n = 15,711*	−0.10	<0.001	−0.13	−0.07

Data Source: National Survey of Children’s Health (NSCH), 2020–2023;

* complete cases;

** Among those with need for care coordination; Models controlled for child sex, race/ethnicity, age, impairments in daily activities, insurance type, gaps in insurance, family poverty level, family structure, household language, highest caregiver education level, and survey year; AME = Average Marginal Effect

Alternative text: The probability of unmet mental health needs was 8 percentage points lower when children received effective care coordination and 11 percentage points lower when providers communicated with school or childcare

## Data Availability

Data is available publicly through the Data Resource Center for Child and Adolescent Health at https://www.childhealthdata.org/learn-about-the-nsch/NSCH.
